# Targeted Genome Editing *via* CRISPR in the Pathogen *Cryptococcus neoformans*

**DOI:** 10.1371/journal.pone.0164322

**Published:** 2016-10-06

**Authors:** Samantha D. M. Arras, Sheena M. H. Chua, Maha S. I. Wizrah, Joshua A. Faint, Amy S. Yap, James A. Fraser

**Affiliations:** 1 Australian Infectious Diseases Research Centre, St Lucia, Queensland, Australia; 2 School of Chemistry & Molecular Biosciences, The University of Queensland, St Lucia, Queensland, Australia; University of Minnesota, UNITED STATES

## Abstract

Low rates of homologous integration have hindered molecular genetic studies in *Cryptococcus neoformans* over the past 20 years, and new tools that facilitate genome manipulation in this important pathogen are greatly needed. To this end, we have investigated the use of a Class 2 CRISPR system in *C*. *neoformans* (formerly *C*. *neoformans* var. *grubii*). We first expressed a derivative of the *Streptococcus pyogenes* Cas9 nuclease in *C*. *neoformans*, and showed that it has no effect on growth, production of virulence factors *in vitro*, or virulence in a murine inhalation model. In proof of principle experiments, we tested the *CAS9* construct in combination with multiple self-cleaving guide RNAs targeting the well-characterized phosphoribosylaminoamidazole carboxylase-encoding *ADE2* gene. Utilizing combinations of transient and stable expression of our constructs, we revealed that functionality of our CRISPR constructs in *C*. *neoformans* is dependent upon the *CAS9* construct being stably integrated into the genome, whilst transient expression of the guide RNA is sufficient to enhance rates of homologous recombination in the *CAS9* genetic background. Given that the presence of the CRISPR nuclease does not influence virulence in a murine inhalation model, we have successfully demonstrated that this system is compatible with studies of *C*. *neoformans* pathogenesis and represents a powerful tool that can be exploited by researchers in the field.

## Introduction

Over the past thirty years the incidence of life-threatening infections caused by opportunistic fungal pathogens has skyrocketed due to the AIDS pandemic and the advent of new immune-suppressing technologies. The majority of these infections are caused by *Candida albicans*, *Aspergillus fumigatus* and *Cryptococcus neoformans*. For those suffering from AIDS, the most dangerous of these pathogens is *C*. *neoformans*; the epidemiology of cryptococcosis mirrors that of Human Immunodeficiency Virus, with a formidable incidence rate in countries in which the burden of HIV is high [1, 2]. Existing in the environment as a ubiquitous saprophyte, the pathogen disseminates *via* the lungs to the central nervous system causing life-threatening meningoencephalitis. Even with the best available treatments, mortality in developed countries can be up to 20%. In less privileged nations where treatment is limited, there is a much poorer patient prognosis, with mortality rates as high as 75% [1–4].

Our understanding of the mechanisms employed by *C*. *neoformans* to cause disease in the human host has been greatly advanced through the development of molecular genetic techniques that have facilitated the investigation of virulence determinants. Yet the low rate of homologous recombination during transformation of this organism remains a major hurdle, impeding efficient analysis of gene function [5–8]. Transformation of *C*. *neoformans* was first achieved in 1990 through electroporation [9], however the technique was not widely adopted due to the instability of transformants and low homologous integration rates. Molecular genetic modifications did not become routine in *C*. *neoformans* research until after the development of a biolistic transformation protocol in 1993 [8]. While this approach is now utilized extensively in the field, it typically only achieves homologous recombination rates of around 10%.

The low rate of homologous integration in *C*. *neoformans* is due largely to the efficiency of Non-Homologous End Joining (NHEJ) driving ectopic integration as the most common fate of transformed DNA. As in other species, removal of a component of the Ku heterodimer that is required for the first step of NHEJ disables this process, resulting in a higher frequency of homologous integration [6]. Unfortunately, *kuΔ* strains present a number of issues; *KU80* expression is upregulated during infection of a human host, and the *ku80Δ* mutant is less successful in a murine competition model of infection [10, 11]. Disrupting NHEJ through the use of *kuΔ* mutants is therefore an inappropriate choice for many studies, and as such these strains have not seen widespread use within the *C*. *neoformans* community.

One alternative to disrupting NHEJ by disabling Ku is to instead enhance the local rate of homologous integration by introducing a double stranded break, thus activating mechanisms that can integrate transforming constructs through homology during the DNA repair process [12]. Over the last three years, an approach that has been successfully employed in numerous organisms is to create targeted double stranded breaks *via* Clustered Regularly Interspaced Short Palindromic Repeat (CRISPR) technology. CRISPR is an adaptive immunity mechanism in prokaryotes [13]. While there are a number of classes of CRISPR systems, they all generally act in three stages. In the first stage, acquisition, the microbe identifies non-self DNA from bacteriophages and other invading genetic elements with the aid of CRISPR associated (Cas) proteins. These identify Protospacer Adjacent Motifs (PAMs), usually the sequence NGG (where N stands for any base), and copy ~24–48 bp upstream of this motif as a “spacer” into a CRISPR locus between two short (~30 bp) palindromic repeats. The sequential addition of new spacers over time generates CRISPR loci that can contain as many as several hundred repeats [13–17]. In the second stage, expression, the CRISPR locus is transcribed, producing pre-crRNA which is cleaved to form crRNAs consisting of a single spacer and an associated repeat. The crRNA then associates with a nuclease; in Class 1 systems this is a multi-subunit complex (Cascade in *Escherichia coli*), in Class 2 it is a single protein (Cas9 in *Streptococcus pyogenes*). In most systems this association is dependent on the crRNA first hybridizing with a complementary *trans*-activating CRISPR RNA (tracrRNA) [18]. In the third and final stage, interference, foreign DNA identical to the crRNA spacer is recognized and cleaved by the nuclease.

First discovered in the bacterium *E*. *coli* and the archaea *Haloferax mediterranei* [19, 20], CRISPR systems have now been identified in a wide range of prokaryotes. Following success in harnessing the activity of CRISPR in prokaryotes [21], a minimal system was engineered for use in eukaryotes. Analyses of a range of CRISPR nucleases identified the simple Class 2 enzyme of *S*. *pyogenes* as effective in human cells following codon optimization and the attachment of an SV40 nuclear localization sequence. Furthermore, experiments with crRNA and tracrRNA revealed these could be fused, enabling targeting of nuclease activity with a guideRNA (gRNA), a single engineered RNA molecule with characteristics of both the crRNA and tracrRNA components.

The utility of the minimal CRISPR-Cas9 system in enabling targeted gene disruption has now been exploited in a diverse range of eukaryotes, including mice and rats [22–25], plants [26–28] and also the fungal species *Saccharomyces cerevisiae* [29], *Schizosaccharomyces pombe* [30], *A*. *fumigatus* [31] and *C*. *albicans* [32]. The successful use of a CRISPR-Cas9 system has also recently been described for *Cryptococcus deneoformans* [33] (formerly *C*. *neoformans* var. *neoformans*), a less clinically prevalent member of the *Cryptococcus* genus [34]. Intriguingly, it was reported that expression of Cas9 in *C*. *neoformans* “exhibits a remarkably negative effect on virulence”, indicating that this important new and widespread molecular tool cannot be employed in the species most commonly associated with life-threatening cryptococcosis.

We have investigated the application of CRISPR in *C*. *neoformans*, creating an expression construct for the *S*. *pyogenes* Cas9 nuclease and generating a strain with it integrated at the well-characterized Safe Haven region of chromosome one [35]. Contrary to the previous report, we have demonstrated that constitutive expression of Cas9 in *C*. *neoformans* has no effect on growth, virulence factor production or the ability to cause disease in a murine inhalation model of infection. Co-transforming the Cas9 strain simultaneously with deletion and self-cleaving gRNA constructs targeting the phosphoribosylaminoamidazole carboxylase-encoding *ADE2* gene dramatically increased the rate of homologous recombination, indicating that this system can be successfully employed in *C*. *neoformans* as an important tool to facilitate targeted modifications of the genome.

## Methods

### Strains and growth conditions

*C*. *neoformans* strains generated in this study are listed in S1 Table and were grown on YPD agar unless stated otherwise. All strains were created in *C*. *neoformans* type strain H99 [36]. Plasmid maintenance and cloning was carried out using the *E*. *coli* strain Mach1 (Life Technologies, USA). Plasmids generated in this study are listed in S2 Table.

### Bioinformatics

Sequence analyses were performed using BLASTp and MacVector 10.0 (MacVector Inc., USA), with sequence alignments generated using ClustalW v1.4. Sequence traces generated at the Australian Genome Research Facility (Australia) were analyzed using Sequencher 4.7 (Gene Codes Corporation, USA).

### Construction of a *C*. *neoformans* strain containing a *CAS9* expression cassette

The *C*. *neoformans CAS9* expression construct was created by combining the *TEF1* (CNAG_06125) *C*. *neoformans* promoter (primers UQ3590 and UQ3591), a human codon optimized, SV40 NLS-containing version of the *S*. *pyogenes CAS9* gene [37] (UQ3592 and UQ3593), and *TEF1* terminator (UQ3594 and UQ3595) by overlap PCR (UQ3590 and UQ3595). All PCR was performed using Phusion High-Fidelity DNA polymerase (New England Biolabs, USA) using H99 genomic DNA as the template for *TEF1* and plasmid p414-*TEF1*p-Cas9-*CYC1* for *CAS9* (Plasmid 43802; Addgene, USA) [29]. Primers are listed in S3 Table. The overlap product was cloned as a NotI/KpnI fragment into the Safe Haven hygromycin B resistance vector pSDMA58 (Plasmid 67942; Addgene, USA) [35] and sequenced. The resulting plasmid pSDMA65 was linearized using BaeI and biolistically transformed into type strain H99, with transformants selected on YPD agar supplemented with 200 μg/mL hygromycin B (Life Technologies, USA) [5]. Integration of the plasmid at the Safe Haven site was confirmed *via* colony PCR [35] and Southern blot using the Safe Haven fragment as a probe (primers UQ2962 and UQ2963), creating strain H99_*CAS9*_.

### Quantitative real-time PCR

Strains H99 and H99_*CAS9*_ were grown in YPD media shaking at 30°C for 16 hr. Cells were collected, and the resulting pellets frozen and lyophilized, with total RNA isolated using TRIzol Reagent (Life Technologies, USA). cDNA was created using the Superscript III First-Strand Synthesis System (Invitrogen, USA) according to the manufacturer’s protocol. Quantitative real-time PCR (qRT-PCR) of *CAS9* (primers UQ3813 and UQ3814) was performed using SYBR Green Supermix (Applied Biosystems, USA) and an Applied Biosystems 7900HT Fast Real Time PCR System with the following cycling conditions: denaturation at 95°C for 10 min, amplification and quantification for 45 cycles at 95°C for 15 sec and 60°C for 1 min, with melting-curve profiling of 95°C for 2 min, 60°C for 15 sec, and 95°C for 15 sec. Relative gene expression was quantified using SDS 1.3.1 (Applied Biosystems, USA) based on the 2^−ΔΔCT^ method [38]. The actin-encoding gene *ACT1* (primers UQ482 and UQ728) was used as a control for normalization. One-way analysis of variance was performed using the unpaired, two-tailed *t*-test in GraphPad Prism Version 7.0 (GraphPad Software, USA). *P*-values of <0.05 were considered statistically significant.

### Phenotypic and virulence factor assays

Fresh cultures of H99 and H99_*CAS9*_ grown in YPD were washed and diluted to OD_600_ 1.0, and 10-fold serially diluted prior to spotting. Melanization assays were performed on solid L-DOPA media supplemented with 10 mM asparagine [39]. Phospholipase B production was visualized on Sabouraud dextrose agar with 8% egg yolk [40], protease production on complete YNB with 1% BSA [41] and urease production on Christensen’s agar [42]. Capsule growth was induced by growth for 24 hr in RPMI 1640 media (Life Technologies, USA) with 2% glucose and 10% fetal bovine serum (Life Technologies, USA) and stained with India Ink (BD Diagnostics, USA). Cells were imaged with a Leica DM2500 microscope and DFC425C camera (Leica, Germany). At least 5 independent images were taken and the relative capsule diameter of 25 cells from each culture determined as described by Zaragoza *et al*. [43]. Capsule diameter was measured relative to cellular diameter using the ruler tool in Adobe Photoshop CS6 (Adobe Systems Incorporated, USA). Experiments were performed in biological triplicate and unpaired, two-tailed *t*-tests were performed in GraphPad Prism Version 7.0 (GraphPad Software, USA) to compare variation between replicates. All assays were performed at both 30 and 37°C.

### Murine inhalation model of cryptococcosis

For murine infection assays, 6-week-old female BALB/c mice (Animal Resources Centre, Australia) were infected by nasal inhalation [44]. For each strain (H99 and H99_*CAS9*_), 10 mice were inoculated with a 50 μL drop containing 5 × 10^5^ *C*. *neoformans* cells. A maximum of 5 mice were housed per IVC cage (Tecniplast, USA) with bed-o’ cobs 1/8” bedding (The Andersons, USA), crink-L’ nest nesting material (The Andersons, USA), and cardboard as environmental enrichment. Mice were provided Rat and Mouse Cubes (Specialty Feeds, Australia) and water *ad libitum*. Each mouse was examined and weighed twice daily for the duration of the experiment, with affected mice euthanized *via* CO_2_ inhalation once body weight had decreased to 80% of pre-infection weight or they exhibited symptoms consistent with infection. Death after CO_2_ inhalation was confirmed by pedal reflex prior to dissection. The brain, lungs, liver, spleen and kidneys were collected, homogenized and plated to determine colony-forming units per gram organ weight. Kaplan-Meier survival curves were plotted using GraphPad Prism 7.0 (GraphPad Software, USA). Significance was analyzed using the log-rank test, while organ burden significance was determined using a one-way ANOVA with Tukey’s multiple comparisons test. *P v*alues of <0.05 were considered significant.

### Ethics statement

This study was carried out in strict accordance with the recommendations in the Australian Code of Practice for the Care and Use of Animals for Scientific Purposes by the National Health and Medical Research Council. The protocol was approved by the Molecular Biosciences Animal Ethics Committee (AEC) of The University of Queensland (AEC approval no. SCMB/439/13/UQ/NHMRC). Infection was performed under methoxyflurane anaesthesia, and all efforts were made to minimize suffering through adherence to the Guidelines to Promote the Wellbeing of Animals Used for Scientific Purposes as put forward by the National Health and Medical Research Council (Australia).

### Construction of *ADE2-targeting* gRNAs

The genome sequence of *ADE2* from strain H99 was imported into MacVector 10.0 (MacVector Inc., USA) and searched for potential PAM-adjacent gRNA seed sequences, which were in turn classified by strand, PAM context, %GC content and uniqueness in the genome. Two gRNA targets were chosen: gRNA1 (CCCGACGGAAGAGGATTTTA; chromosome 6 coordinates 679,760–679,779) and gRNA2 (AGCCTGATGCCCATGCAGAC; chromosome 6 coordinates 680,568–680,587).

The gRNA expression constructs were synthesised as 1,514 bp gBlock fragments (IDT, USA). Each consisted of a XhoI site, the *C*. *neoformans ACT1* promoter (chromosome 1 coordinates 1,242,235–1,243,067), the first 6 bp of the chosen *ADE2* gRNA target region, the hammerhead ribozyme [45], the chosen 20 bp *ADE2* gRNA target region, the HDV ribozyme [45], the *TRP1* terminator (chromosome 9 coordinates 1,023,924–1,024,166) and finally a SacI site. Both gBlocks were amplified (primers UQ18 and UQ19) using Phusion High-Fidelity DNA polymerase (New England Biolabs, USA), the products digested with XhoI and SacI, and ligated into XhoI- and SacI-digested *NEO* resistance plasmid pJAF1 [46], creating gRNA1 plasmid pSDMA64 and gRNA2 plasmid pASY1, and into the Safe Haven vector pSDMA25 (Plasmid 67940; Addgene, USA) [35] creating pSDMA66 (gRNA1) and pSDMA67 (gRNA2). All plasmids were sequenced to ensure they were error free. pSDMA66 and pSDMA67 were subsequently linearized with BaeI and biolistically transformed into type strain H99 [5]. Stable transformants were selected on YPD supplemented with 100 μg/mL nourseothricin (Werner Bioagents, Germany). Integration of the plasmids at the Safe Haven site was confirmed *via* colony PCR [35] and Southern blot using the Safe Haven fragment as a probe (primers UQ2962 and UQ2963), creating the strains H99_*gRNA1*_ and H99_*gRNA2*_.

### Construction of *ADE2* mutant strains

A deletion construct for the *ADE2* gene was generated as per Arras *et al*. [35]. Briefly, the construct was prepared using overlap PCR, employing primers UQ1439 and UQ1442 to join the 994 bp *ADE2* 5’ region (primers UQ1439 and UQ1440), the G418 resistance marker *NEO* (UQ2808 and UQ2809) and the *ADE2* 982 bp 3’ region (UQ1441 and UQ1442). H99 genomic DNA was used as the template for *ADE2*, and the plasmid pJAF1 for *NEO* [46]. *C*. *neoformans* transformations were carried out *via* biolistic particle delivery as previously described [5] onto media containing 100 μg/mL G418 (Sigma, USA). Correct *ade2Δ* integrants were initially identified *via* their pink coloration on YPD media and adenine auxotrophy on YNB media. Representative pink transformants were selected for Southern blot analyses to validate correct integration of the *ADE2* deletion cassette using the UQ1439 and UQ1442 PCR product as a probe. Only transformations that resulted in 10 or more colonies were included in subsequent analyses due to larger numbers providing a greater level of precision. To determine significance of correct integration rates between conditions, ANOVA followed by Tukey’s HSD tests were completed in GraphPad Prism Version 7.0 (GraphPad Software, USA). *P*-values of <0.05 were considered statistically significant. The absence of the gRNA plasmids following co-transformation with the *ade2*::*NEO* construct was verified *via* Southern blot, using the UQ234 and UQ235 PCR product generated from plasmid pSDMA64 as a probe.

## Results

### Creating a *CAS9* expression construct for *C*. *neoformans*

**Fig 1:**
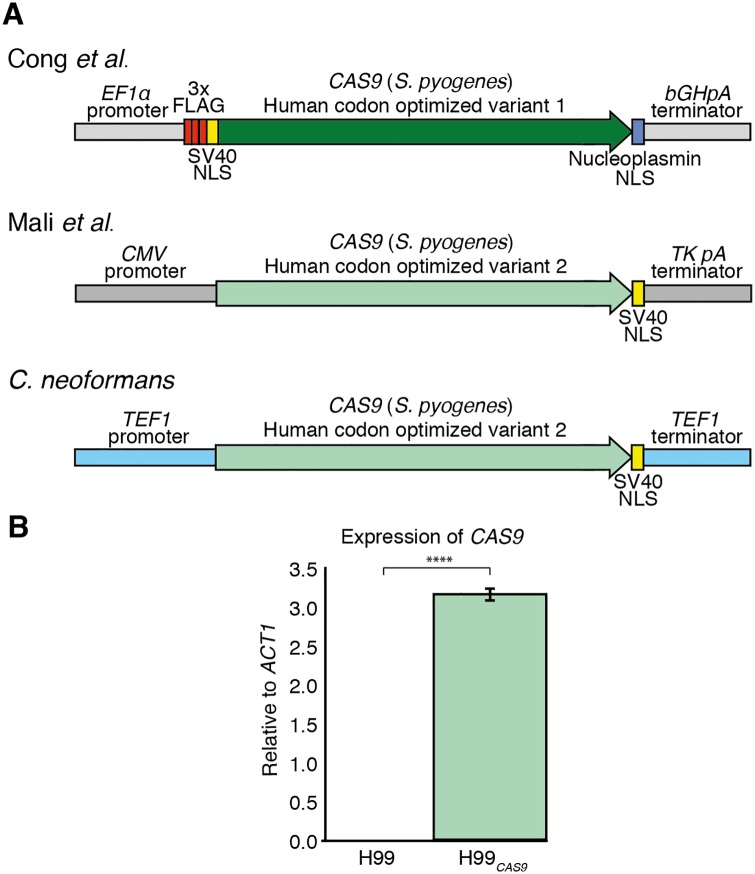
Expressing Cas9 in *C*. *neoformans*. **A.** Comparison of the *CAS9* expression constructs from human [37, 47] and *C*. *neoformans*. **B.** Transcript abundance of *CAS9* in H99 and H99_*CAS9*_ relative to *ACT1*. Values show mean, error bars show S.E.M.

The utility of CRISPR as a molecular tool in eukaryotes was first proven in human cell lines, with two groups developing constructs to express the Class 2 Type II Cas9 from *S*. *pyogenes* (Fig 1A) [37, 47]. To begin our studies in *C*. *neoformans*, we investigated these humanized *CAS9* constructs as a starting point. Comparing the *CAS9* coding regions of the humanized constructs revealed only 81.1% nucleotide identity, with over half of the codons differing by at least one nucleotide; neither publication described the method of codon optimization for humans, but they clearly used very different criteria. Of the two, the construct from Mali *et al*. [37] had both codon usage and GC content more closely aligned to that published for *C*. *deneoformans* [48]. These humanized *CAS9* constructs also differ in their complexity. Both constructs contain the monopartite SV40 large T-antigen NLS for nuclear localization, but the Cong *et al*. [47] construct also includes a triplicate FLAG tag, and the bipartite *Xenopus laevis* nucleoplasmin NLS (Fig 1A). As we could not predict the consequences in *C*. *neoformans* of introducing each additional construct element, we considerred the simplicity of the Mali *et al*. construct to be more suitable for our intended purpose. Furthermore, the Mali *et al*. construct had been successfully employed in *S*. *cerevisiae*, demonstrating it can function in fungi [29].

To adapt the Mali *et al*. construct for use in *C*. *neoformans*, we first selected a promoter and terminator that would enable expression in this pathogen. Based on the success of DiCarlo *et al*. who used the elongation factor 1-alpha (*TEF1*) promoter to express *CAS9* in *S*. *cerevisiae* [29], we opted to use the promoter of the *C*. *neoformans TEF1* homolog. The *C*. *neoformans TEF1* 5’ UTR begins 500 bp upstream of the ATG and includes an intron. In order to include this long UTR and associated regulatory elements, we PCR amplified the promoter as a 1,256 bp fragment, combined it *via* overlap PCR with the *CAS9-SV40* ORF and the *TEF1* terminator region, and subcloned this product into the pSDMA58 Safe Haven vector for targeting to chromosome 1 (Fig 1A) [35]. The construct was then integrated into the genome of *C*. *neoformans* type strain H99 to create strain H99_*CAS9*_. Following confirmation of successful integration of our *CAS9* construct at the correct genomic location, *CAS9* expression was confirmed using qRT-PCR (Fig 1B).

### Expressing Cas9 in *C*. *neoformans* does not influence growth or virulence factor production

**Fig 2:**
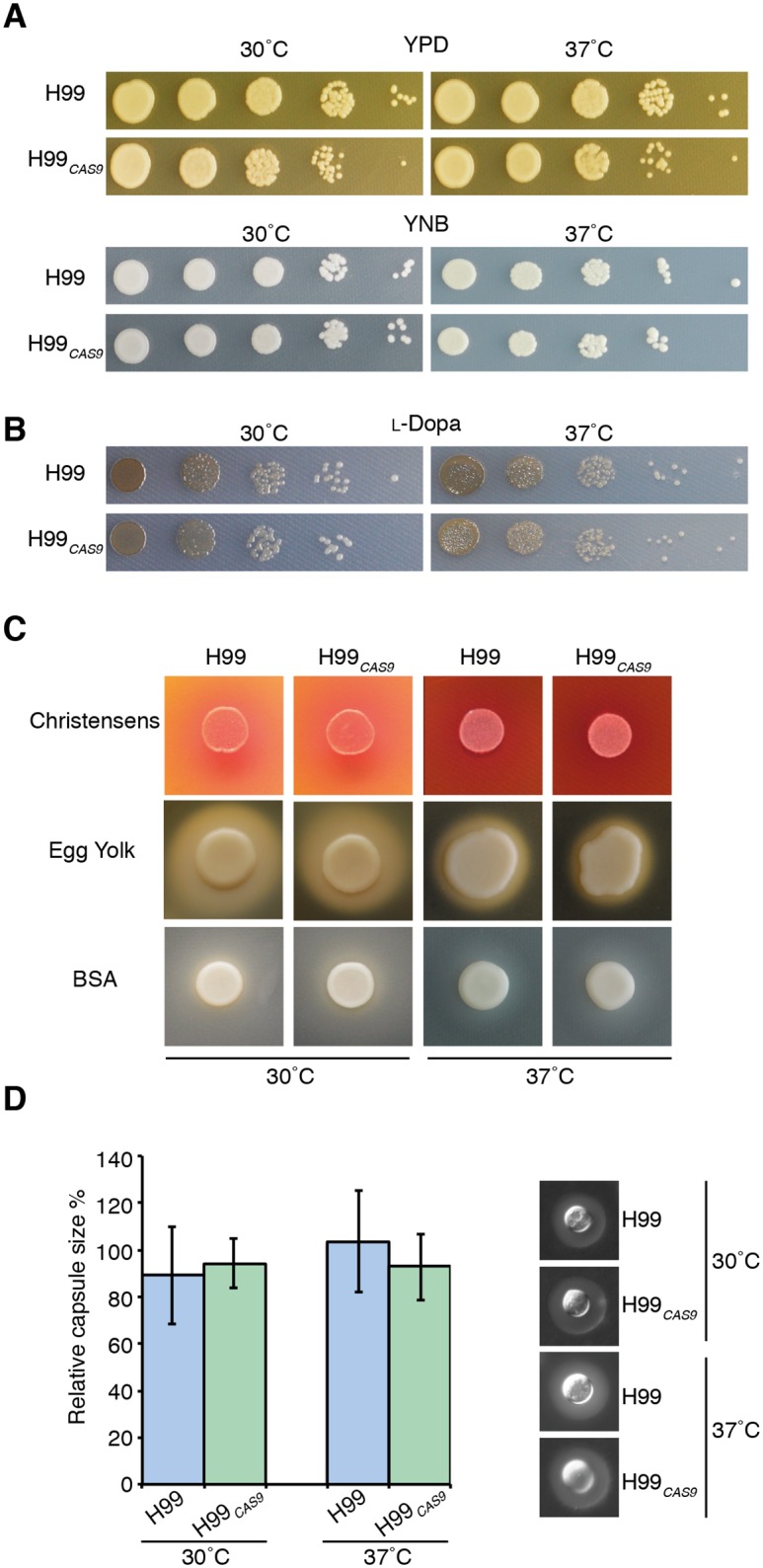
The *C*. *neoformans* Cas9 strain is indistinguishable from wild-type in growth or virulence production assays. H99 and H99_*CAS9*_ were compared for growth on YPD and YNB (**A**), melanin production on L-DOPA (**B**), urease production on Christensen’s agar, phospholipase production on egg yolk agar, protease production BSA agar (**C**) and capsule production in RPMI media (**D**). All assays were visualized at 48 hr at 30 and 37°C excluding growth on egg yolk agar, which was visualized at 96 hr, and capsule, visualized at 24 hr.

For CRISPR to be useful in *C*. *neoformans*, *CAS9* expression must not influence normal growth or any virulence-associated characteristics, such as growth at body temperature or production of melanin, urease, phospholipase, protease and capsule. Growth of the *CAS9* strain H99_*CAS9*_ was indistinguishable from wild-type at 30°C or at 37°C (human body temperature) on both rich YPD and synthetic YNB media (Fig 2A). Strains were again indistinguishable for melanin production as measured on l-DOPA medium, producing equivalent pigmentation at both temperatures (Fig 2B). Indeed, there was no measurable distinction between the wild-type and *CAS9* strains in any of the *C*. *neoformans* virulence factor *in vitro* assays performed at either 30 or 37°C; H99 and H99_*CAS9*_ were the same for urease production on Christensen’s agar, phospholipase production on egg yolk agar, protease production on BSA agar (Fig 2C), and capsule production in RPMI media (Fig 2D). In short, we saw no evidence that *TEF1* promoter-driven heterologous expression of the humanized, SV40 NLS-containing *S*. *pyogenes CAS9* construct influenced *C*. *neoformans* growth or virulence factor production in any way.

### Expressing Cas9 in *C*. *neoformans* does not influence the progression of disease

**Fig 3:**
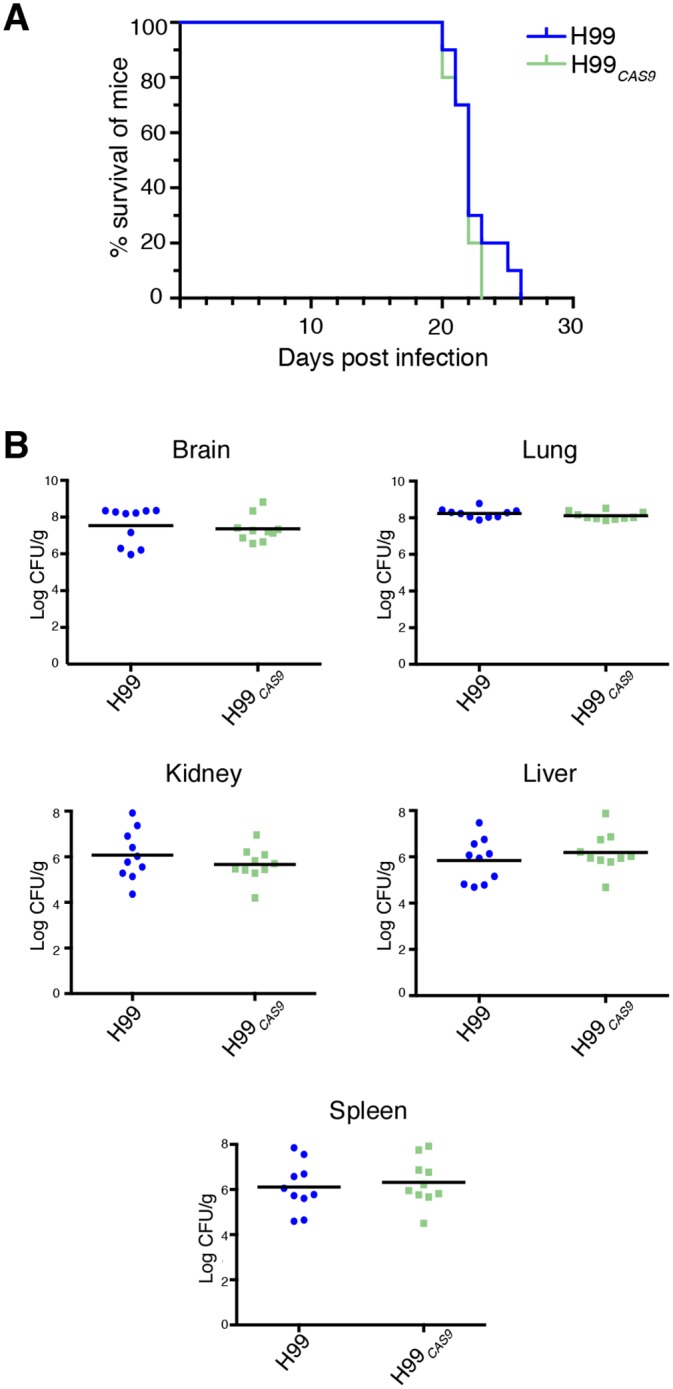
Virulence of H99_CAS9_ is indistinguishable from wild-type in a mouse model. **A.** No significant difference was found between H99 and H99_*CAS9*_ in a murine inhalation model of virulence. **B.** No significant difference was observed in fungal organ burden of mice infected with H99 and H99_*CAS9*_.

Given that the *CAS9*-expressing H99_*CAS9*_ strain showed no detectable differences from wild-type in any *in vitro* analyses, we progressed our studies to animal infections. Mice infected intranasally with the *CAS9* strain were indistinguishable from mice infected with wild-type, with both cohorts succumbing to infection within the typical timespan of three weeks (Fig 3A). To gain a deeper insight into the progression of infection, and to determine whether the presence of the *CAS9* construct alters the tropism of the pathogen, a post-mortem analysis of fungal organ burden of the brain, lungs, kidneys, liver and spleen was performed. Again, the H99_*CAS9*_ strain was indistinguishable from its wild-type parent (Fig 3B). Together with the *in vitro* data, these results indicated that Cas9 does not influence growth or virulence, and could therefore be employed in molecular studies of *C*. *neoformans* pathogenesis.

### Creation of gRNA constructs designed to target *ADE2*

To function, CRISPR requires the expression of a nuclease and the synthesis of an RNA molecule to guide the nuclease activity. To investigate the utility of our *CAS9* construct, we therefore needed to generate appropriate guide RNAs. However, transcribing these molecules in eukaryotes is problematic; most promoters are transcribed by RNA polymerase II, which performs 5’ capping and polyadenylation that abolishes gRNA function. One solution is the use of an RNA polymerase III promoter such as *U3*, *U6* or *SNR52*, however these add a 5’ guanine to the transcript, limiting usable PAMs to those that have a corresponding G at the 5’ end of the target sequence [37]. Furthermore, the precise start point of transcription must be known, and to our knowledge no RNA polymerase III promoters have been well characterized in *C*. *neoformans*.

The difficulties associated with eukaryotic gRNA synthesis have been addressed by Gao and Zhao [45], who devised a strategy where an RNA polymerase II promoter is used to transcribe a synthetic gene consisting of the Hammerhead and Hepatitis Delta Virus ribozymes flanking the desired gRNA sequence. Following transcription, the 5’ capped, polyadenylated transcript undergoes self-cleavage, liberating an unmodified gRNA molecule. We followed this scheme, employing the *ACT1* promoter / *TRP1* terminator combination already used in a variety of *C*. *neoformans* constructs [35, 46, 49]. For the target sequence, we chose the well-characterized phosphoribosylaminoimidazole carboxylase-encoding *ADE2* gene as a proof of principle example; loss of *ADE2* results in the easily observable phenotypes of adenine auxotrophy and pink coloration due to accumulation of the purine biosynthetic intermediate P-ribosylaminoimidazole [50].

**Fig 4:**
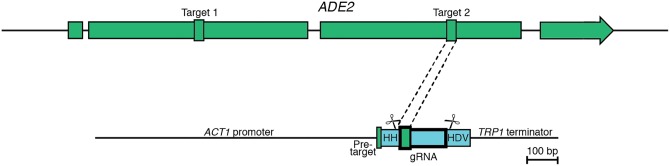
gRNA constructs targeting *C*. *neoformans ADE2*. The target sites for gRNA1 and gRNA2 within the *ADE2* gene are indicated; the gRNA construct shown is for gRNA2. HH, Hammerhead ribozyme; HDV, Hepatitis Delta Virus ribozyme. Ribozyme cleavage sites are represented as scissors.

Searching both strands of the *ADE2* gene, we identified 275 canonical PAM sequences (NGG). To eliminate less favorable gRNA targets, we adopted the guidelines of Wang and colleagues [51], rejecting the 148 (54%) on the transcribed strand, and a further 41 (15%) where the PAM sequence was followed by a guanine residue; both of these characteristics have been shown to reduce gRNA effectiveness. Of the remaining 82, those with target sequences well within the suggested 40–80% GC content were prioritized [52]. Based on these criteria, we chose two targets, one approximately a third of the way through the *ADE2* gene (gRNA1) (448 bp from the ATG), and a second approximately two thirds of the way through (gRNA2) (1256 bp from the ATG) (Fig 4).

The final engineered gRNA constructs contain the *ACT1 C*. *neoformans* promoter, an *ADE2* pre-target region of 6 bp required for Hammerhead ribozyme function, the Hammerhead ribozyme, the 20 bp target sequence, the conserved non-target elements of the gRNA molecule, the HDV ribozyme and lastly the *TRP1* terminator. We subcloned these products into the pSDMA25 Safe Haven vector for targeting to chromosome 1. The Safe Haven constructs were then integrated into the genome of *C*. *neoformans* type strain H99 to create strains H99_*gRNA1*_ and H99_*gRNA2*_.

### Co-transformation with plasmid-borne *CAS9* and gRNA constructs does not enhance *ADE2* deletion frequency

The Cas9/gRNA complex introduces double stranded breaks at the gRNA target sequence, which triggers activation of repair mechanisms and enhances homologous recombination. To determine if this process could increase the rate of targeted gene deletion in *C*. *neoformans*, we biolistically co-transformed an *ade2*::*NEO* deletion construct with a combination of CRISPR constructs on circular plasmids—either *CAS9* plus gRNA1, or *CAS9* plus gRNA2. By employing G418 media, there was no selective pressure to integrate or maintain the CRISPR plasmids; furthermore, unlike *C*. *deneoformans*, *C*. *neoformans* is unable to stably maintain episomal constructs, typically losing constructs within a few generations [7, 9].

**Fig 5:**
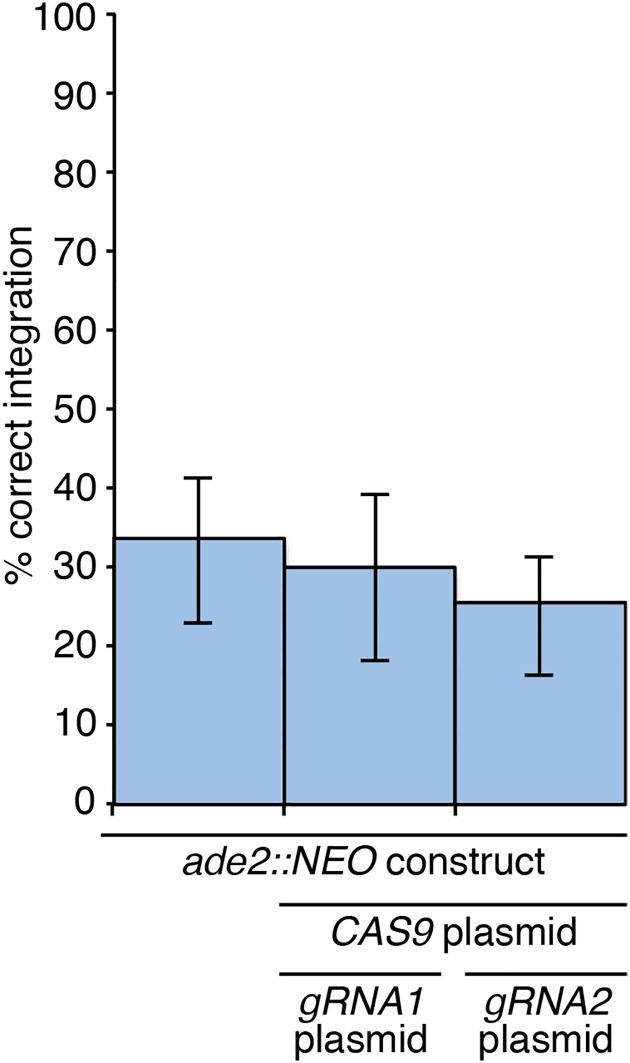
Co-transformations combining the *ade2*::*NEO* deletion construct with plasmid-borne Cas9 and gRNA constructs does not enhance the frequency of *ADE2* deletion. No increase in the rate of homologous recombination was observed between transformations with a deletion construct only and transformations with the deletion construct, Cas9 plasmid and gRNA plasmid present. Values show mean, error bars show S.E.M.

In our control transformation with just the *ade2*::*NEO* construct, the frequency of gene deletion was 33% (Fig 5), consistent with our previous investigations that have shown that this locus is more amenable than most in *C*. *neoformans* to deletion (35). Importantly, the co-transformation of the *ade2*::*NEO* construct with both the Cas9 plasmid and a gRNA plasmid showed no significant change in integration frequency, with the *ADE2* deletion construct integrating at the correct position 30% of the time for gRNA1 and 26% for gRNA2 (Fig 5).

### CRISPR in *C*. *neoformans* leads to increased homologous integration rates

**Fig 6:**
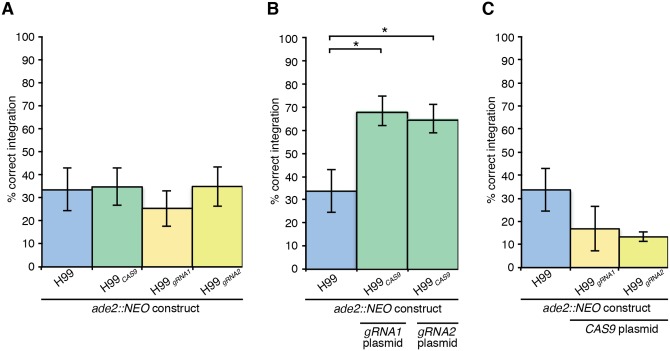
Deleting *ADE2* with the aid of a CRISPR-Cas9 system. **A.** Transformations where only one part of the CRISPR system was included to determine if constitutive expression of individual components had any effect on the rate of homologous integration at the *ADE2* locus. **B.** Transformations where the recipient strain is stably expressing *CAS9* at the Safe Haven site, with gRNA plasmids co-transformed with the *ade2Δ* construct. **C.** Transformations where the recipient strain is stably expressing a gRNA construct at the Safe Haven site, with the *CAS9* plasmid co-transformed with the *ade2Δ* construct. For all graphs, values show mean, error bars show S.E.M and * = P<0.05.

Given that the inclusion of our circular, unselected CRISPR plasmids during co-transformation had no influence on the rate of *ADE2* deletion, we next investigated the effect of having each of these elements stably expressed in the genome from the Safe Haven site during transformation with the *ade2*::*NEO* construct. The rate of *ADE2* deletion was equivalent in H99, H99_*CAS9*_, H99_*gRNA1*_ and H99_*gRNA2*_ as expected; with only one of the elements of the CRISPR system present, no increase was expected (Fig 6A). Moreover, we saw no evidence of negative effects from stable expression of single CRISPR elements on the transformation process.

Once we had established that each CRISPR element alone did not influence the rate of *ADE2* deletion, we employed the Safe Haven H99_*CAS9*_ strain in co-transformations with the *ade2*::*NEO* and gRNA constructs. Unlike the previous experiments, we observed a substantial increase in the frequency of deletion of *ADE2*, raising to 70% (Fig 6B); under appropriate conditions, CRISPR functions in *C*. *neoformans* to enhance the rate of gene deletion without requiring integration of the gRNA plasmid (S1 Fig). Given our CRISPR success in H99_*CAS9*_, we next investigated the effect of employing the Safe Haven H99_*gRNA1*_ and H99_*gRNA2*_ strains in co-transformations with the *ade2*::*NEO* and *CAS9* constructs. In stark contrast to the H99_*CAS9*_ success, the frequency of *ADE2* deletion was not statistically different to the H99 control in these experiments (Fig 6C). Together, these data confirm that we have successfully developed a functioning CRISPR system in *C*. *neoformans* by combining a strain stably expressing *CAS9* with transient expression of self-cleaving gRNA constructs.

## Discussion

The current tools and methodologies available for study of *C*. *neoformans* have significantly advanced our understanding of the mechanisms influencing virulence in this important pathogen. However, routine gene deletions can still be difficult to achieve, significantly hindering molecular genetic studies. Gene deletion through homologous recombination can be improved by several already published approaches. The low deletion rates already achieved are dependent on the use of homologous flanks in the transforming construct of around 1 kb, much longer than the size required in *S*. *cerevisiae*. Increasing these further may increase integration rates, however this benefit would be offset by introducing difficulties in PCR amplification and cloning [53]. Another approach is to use *kuΔ* strains; while they can increase the rates of recombination to nearly 100% [6, 7], more recent publications have indicated that the Ku proteins may be playing a role during infection of a human host, and the loss of this protein results in altered virulence in mice [10, 11]. These data indicate that it could be problematic to use Ku mutants for creating gene deletions for the study of pathogenesis in this species, and as a result these strains are rarely utilized.

Unlike the strategies described above, CRISPR avoids interrupting NHEJ, as the Cas9 nuclease is directed to its target with a guide RNA. In this study, we have specifically demonstrated the ability of this system in the context of the *ADE2* locus, increasing homologous integration rates to 70%. In the case of our constructs, this increase in homologous recombination was seen only when Cas9 was stably expressed following integration of the cassette into the genome, not when it was introduced on a non-integrative plasmid. We hypothesize that this is likely due to the construct not expressing the Cas9 nuclease to sufficient levels in time to influence the fate of transforming DNA before the plasmid is lost by the cell.

Importantly, and in stark contrast to recently published work [33], we have found no evidence that constitutive expression of Cas9 in *C*. *neoformans* affects normal growth, *in vitro* production of virulence factors, or the progression of disease in a murine model. However, there were some marked differences in the experimental approaches we employed which may explain this discrepancy. Wang and colleagues utilized the human codon-optimized *CAS9* construct of Cong *et al*. [47], whilst we used the equivalent construct of Mali *et al*. [37]; these only show 81.1% nucleotide identity, and it is possible that the transcript of the Cong construct may contain cryptic intron splice sites recognized by *C*. *neoformans*, or the choice of codons may be highly disfavoured. Furthermore, the *C*. *deneoformans* construct employed a bovine growth hormone terminator, which may function differently in *C*. *neoformans*, and a different promoter, which may express Cas9 at a level that makes it toxic to this species. Finally, the *C*. *deneoformans* Cas9 is fused not only to the SV40 NLS, but to a triplicate FLAG tag and the nucleoplasmin NLS as well. While FLAG has been used in *C*. *neoformans* by others [54, 55], we could find no record of the nuceloplasmin NLS being employed; perhaps it is this element that is toxic. We cannot be certain which of these explanations, if any, are the reason for the differences in this work and that published for *C*. *deneoformans*. Whatever the case, Wang and colleagues obtained excellent results with their construct in *C*. *deneoformans*, just as we have with our own construct in *C*. *neoformans*.

The CRISPR strain described here now joins the *kuΔ* mutants in providing researchers with a second potentially useful background that can be used to facilitate molecular genetic alterations in *C*. *neoformans*. The *kuΔ* strains are straightforward to use, and can be easily transformed with the construct of choice; however, the phenotype associated with the *ku80Δ* mutation is continually present unless outcrossed. The H99_*CAS9*_ strain is more difficult to work with, requiring the creation of a gRNA in addition to the construct being transformed, but this background has the benefit of being an otherwise wild-type strain, with the heterologously expressed *S*. *pyogenes* nuclease inactive unless an appropriate guide molecule is provided. While untested here, we also postulate that this system could be used not only for creating gene deletions by providing a construct but also through the introduction of mutations in the absence of a template.

In summary, we have demonstrated the first proof of principle that CRISPR can be employed as a tool for high efficiency gene disruption in *C*. *neoformans*, holding significant potential for progressing the state of genetic research in this organism and consequently understanding its pathogenicity.

## Supporting Information

S1 Fig Southern blot confirming absence of gRNA plasmids in strains generated through co-transformations employing the *ade2*::*NEO* construct with a gRNA plasmid. All strains were digested with PstI, and probed with the *ACT1* promoter and *TRP1* terminator regions common to the *ACT1* and *TRP1* loci, the gRNA plasmid, the hyg resistance cassette and the G418 resistance cassette. The expected bands as follows: *ACT1* promoter in the native location, 5,715 bp; *ACT1* and *TRP1* associated with the *HYG* resistance marker for integration of *CAS9*: 2,555 and 4,762 bp; *ACT1* and *TRP1* associated with the *NEO* marker for *ade2Δ*: 3,756 and 1,365 bp. If the gRNA plasmid was present, a single additional 4,053 bp would be expected; only one strain showed unexpected bands, however these are likely associated with abnormal integration of the *ade2*::*NEO* construct at the *ADE2* locus, as the strain was an adenine auxotroph, and obtaining unusual transformants is common when biolistically transforming *C*. *neoformans*.
(TIF)

S1 Table Strains used in this study. (DOCX)

S2 Table Plasmids used in this study. (DOCX)

S3 Table Primers used in this study. (DOCX)
