# Supplementary figures and images for: Targeted Genome Editing via CRISPR in the Pathogen Cryptococcus neoformans
Source: PLoS One. 2016 Oct 6;11(10):e0164322. doi: 10.1371/journal.pone.0164322 (PMC5053423; doi:10.1371/journal.pone.0164322)

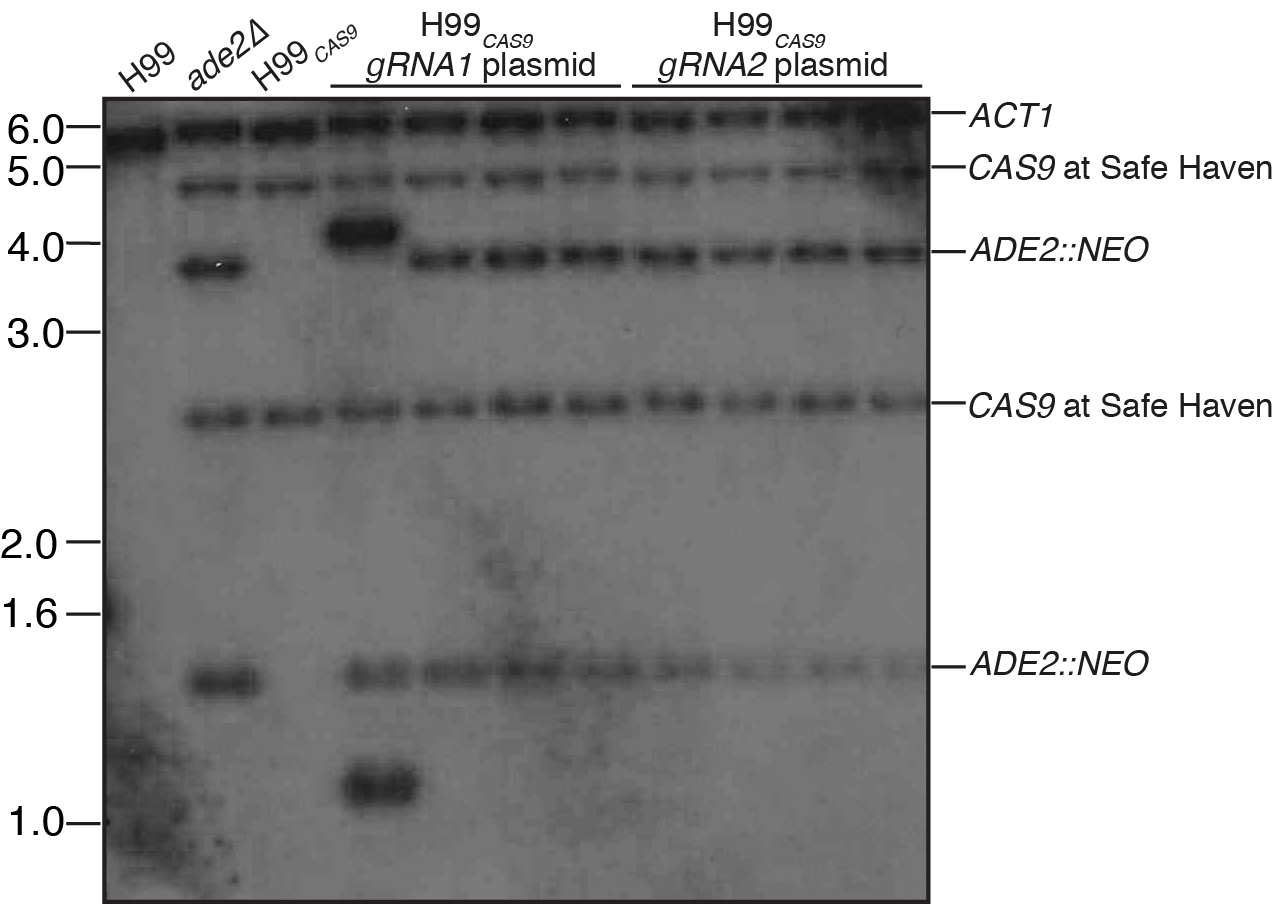

Supplement: S1 Fig — All strains were digested with PstI, and probed with the ACT1 promoter and TRP1 terminator regions common to the ACT1 and TRP1 loci, the gRNA plasmid, the hyg resistance cassette and the G418 resistance cassette. The expected bands as follows: ACT1 promoter in the native location, 5,715 bp; ACT1 and TRP1 associated with the HYG resistance marker for integration of CAS9: 2,555 and 4,762 bp; ACT1 and TRP1 associated with the NEO marker for ade2Δ: 3,756 and 1,365 bp. If the gRNA plasmid was present, a single additional 4,053 bp would be expected; only one strain showed unexpected bands, however these are likely associated with abnormal integration of the ade2::NEO construct at the ADE2 locus, as the strain was an adenine auxotroph, and obtaining unusual transformants is common when biolistically transforming C. neoformans. (TIF) [file pone.0164322.s001.tif]
