# Supplementary material for: Targeted Genome Editing via CRISPR in the Pathogen Cryptococcus neoformans
Source: PLoS One. 2016 Oct 6;11(10):e0164322. doi: 10.1371/journal.pone.0164322 (PMC5053423; doi:10.1371/journal.pone.0164322)
Supplement: S1 Table — (DOCX) [file pone.0164322.s002.docx]

**Supplementary Table 1: Fungal strains used in this study**

| Strain | Genotype | Original source |
| --- | --- | --- |
| H99 | Wild-type | [36] |
| H99*_CAS9_* | *CAS9* cassette at Safe Haven | This study |
| H99*_gRNA2_* | *gRNA1* at Safe Haven | This study |
| H99*_gRNA1_* | *gRNA2* at Safe Haven | This study |
