# Supplementary material for: Targeted Genome Editing via CRISPR in the Pathogen Cryptococcus neoformans
Source: PLoS One. 2016 Oct 6;11(10):e0164322. doi: 10.1371/journal.pone.0164322 (PMC5053423; doi:10.1371/journal.pone.0164322)
Supplement: S2 Table — (DOCX) [file pone.0164322.s003.docx]

**Supplementary Table 2: Plasmids used in this study**

| Strain | Description | Original source |
| --- | --- | --- |
| p414-*TEF1*p-Cas9-*CYC1* | *CAS9* vector | [29] |
| pSDMA58 | *HYG* vector for targeted integration at Safe Haven | [35] |
| pSDMA65 | *C. neoformans CAS9* expression construct | This study |
| pJAF1 | *NEO* resistance cassette vector | [46] |
| pSDMA64 | *gRNA1* in pJAF1 | This study |
| pASY1 | *gRNA2* in pJAF1 | This study |
| pSDMA25 | *NAT* vector for targeted integration at Safe Haven | [35] |
| pSDMA66 | *gRNA1* in pSDMA25 | This study |
| pSDMA67 | *gRNA2* in pSDMA25 | This study |
