# Supplementary material for: Targeted Genome Editing via CRISPR in the Pathogen Cryptococcus neoformans
Source: PLoS One. 2016 Oct 6;11(10):e0164322. doi: 10.1371/journal.pone.0164322 (PMC5053423; doi:10.1371/journal.pone.0164322)
Supplement: S3 Table — (DOCX) [file pone.0164322.s004.docx]

**Supplementary Table 3: Primers used in this study**

| Primer ID | NAME | Sequence |
| --- | --- | --- |
| UQ3590 | *TEF1* promoter upper | ATGTACGCGGCCGCCATTCTATGCTATACGGTACA |
| UQ3591 | *TEF1* promoter lower | ATGCTGTACTTCTTGTCCATTTTGAAGTTTTCTGTGGAGAT |
| UQ3592 | *CAS9* ORF upper | ATCTCCACAGAAAACTTCAAAATGGACAAGAAGTACAGCAT |
| UQ3593 | *CAS9* ORF lower | AAGGGTCACTACATAAGTAATCACACCTTCCTCTTCTTCT |
| UQ3594 | *TEF1* terminator upper | AGAAGAAGAGGAAGGTGTGATTACTTATGTAGTGACCCTT |
| UQ3595 | *TEF1* terminator lower | GTACATGGTACCCGTTCAATCTCATAGTCTTCT |
| UQ482 | *ACT1* qRT PCR upper | CCTACAACTCTATCATGAAGTGTGATCTC |
| UQ728 | *ACT1* qRT PCR lower | TCTGCATACGGTCGGCAATAC |
| UQ3813 | *CAS9* qRT PCR upper | AAGGCTGACTTGCGGTTGA |
| UQ3814 | *CAS9* qRT PCR lower | AGTGTCCCCGAAATTTGATCAT |
| UQ18 | M13F | GTAAAACGACGGCCAG |
| UQ19 | M13R | CAGGAAACAGCTATGAC |
| UQ1439 | *ADE2* 5’ deletion construct upper | GAGTTAAAGTGTCGATGGCAG |
| UQ1440 | *ADE2* 5’ deletion construct lower | CCAGCTCACATCCTCGCAGCTTTGCTACAAGGGGTGCGGATG |
| UQ2808 | *ADE2* *NEO* deletion construct upper | CATCCGCACCCCTTGTAGCAAAGCTGCGAGGATGTGAGCTGG |
| UQ2809 | *ADE2 NEO* deletion construct lower | AGTTACAGATATCGCACCAATGGTTTATCTGTATTAACACGG |
| UQ1441 | *ADE2* 3’ deletion construct upper | CCGTGTTAATACAGATAAACCATTGGTGCGATATCTGTAACT |
| UQ1442 | *ADE2* 3’ deletion construct lower | CGCTTAGGACAAGAGAGGCTA |
| UQ234 | EZoverlap 1 | GCTGCGAGGATGTGAGCTGGAGAGCG |
| UQ235 | EZoverlap 2 | GGTTTATCTGTATTAACACGGAAGAGATGTAG |
